# A *Rb1* promoter variant with reduced activity contributes to osteosarcoma susceptibility in irradiated mice

**DOI:** 10.1186/1476-4598-13-182

**Published:** 2014-08-04

**Authors:** Michael Rosemann, Iria Gonzalez-Vasconcellos, Tanja Domke, Virginija Kuosaite, Ralf Schneider, Markus Kremer, Jack Favor, Michaela Nathrath, Michael J Atkinson

**Affiliations:** 1 Institute of Radiation Biology, Helmholtz-Center Munich, National Research Centre for Health and Environment, Ingolstadter Landstrasse 1, D-85764 Neuherberg, Germany; 2 Institute of Pathology, Helmholtz-Center Munich, National Research Centre for Health and Environment, Ingolstadter Landstrasse 1, D-85764 Neuherberg, Germany; 3 Institute for Experimental Genetics, Helmholtz-Center Munich, National Research Centre for Health and Environment, Ingolstadter Landstrasse 1, D-85764 Neuherberg, Germany; 4 Institute of General Pathology and Pathological Anatomy, Technical University Munich, Ismanninger Strasse 22, D-81675 Munich, Germany; 5 Institute for Human Genetics, Helmholtz-Center Munich, National Research Centre for Health and Environment, Ingolstadter Landstrasse 1, D-85764 Neuherberg, Germany; 6 Clinical Cooperation Group Osteosarcoma, Munich, Germany; 7 Clinic for Paediatric Oncology, Technical University Munich, Ismanninger Strasse 22, D-81675 Munich, Germany; 8 Clinic for Radiation Oncology, Technical University Munich, Ismanninger Strasse 22, D-81675 Munich, Germany

**Keywords:** Tumor susceptibility, Germline polymorphism, Promoter activity, Radiation-induced, Bone tumor, Mouse model

## Abstract

**Background:**

Syndromic forms of osteosarcoma (OS) account for less than 10% of all recorded cases of this malignancy. An individual OS predisposition is also possible by the inheritance of low penetrance alleles of tumor susceptibility genes, usually without evidence of a syndromic condition. Genetic variants involved in such a non-syndromic form of tumor predisposition are difficult to identify, given the low incidence of osteosarcoma cases and the genetic heterogeneity of patients. We recently mapped a major OS susceptibility QTL to mouse chromosome 14 by comparing alpha-radiation induced osteosarcoma in mouse strains which differ in their tumor susceptibility.

**Methods:**

Tumor-specific allelic losses in murine osteosacoma were mapped along chromosome 14 using microsatellite markers and SNP allelotyping. Candidate gene search in the mapped interval was refined using PosMed data mining and mRNA expression analysis in normal osteoblasts. A strain-specific promoter variant in *Rb*1 was tested for its influence on mRNA expression using reporter assay.

**Results:**

A common *Rb1* allele derived from the BALB/cHeNhg strain was identified as the major determinant of radiation-induced OS risk at this locus. Increased OS-risk is linked with a hexanucleotide deletion in the promoter region which is predicted to change WT1 and SP1 transcription factor-binding sites. Both *in-vitro* reporter and *in-vivo* expression assays confirmed an approx. 1.5 fold reduced gene expression by this promoter variant. Concordantly, the 50% reduction in *Rb1* expression in mice bearing a conditional hemizygous *Rb1* deletion causes a significant rise of OS incidence following alpha-irradiation.

**Conclusion:**

This is the first experimental demonstration of a functional and genetic link between reduced *Rb1* expression from a common promoter variant and increased tumor risk after radiation exposure. We propose that a reduced *Rb1* expression by common variants in regulatory regions can modify the risk for a malignant transformation of bone cells after radiation exposure.

## Background

The relatively low incidence of osteosarcoma (OS) in the general population increases dramatically in cases of high–penetrance cancer syndromes such as Li-Fraumeni, familial retinoblastoma or Werner- and Rothmund-Thompson syndrome [1]. Aggregation of osteosarcoma in families without evidence of an underlying tumor syndrome is rare, and it is possible that this is due to a chance observation or shared environmental risk factor [2-4]. Nevertheless, association between osteosarcoma and other tumor types in relatives does occur [5,6], suggesting that undiscovered germline factors may contribute to osteosarcoma risk. The existence of congenital, low-penetrance tumor predisposition associated with OS in non-syndromic patients was also concluded in a study of 938 Dutch osteosarcoma patients, in which a 2.4 fold increased risk of developing an additional tumor in another organ was found [7]. In the case of Li-Fraumeni and congenital retinoblastoma syndromes, two high-penetrance cancer-prone conditions with a predisposition for OS, a subset of the affected patients had not been found to carry germline mutations in the prototypical TP53 gene [8] or in the RB1 coding sequence [9]. It is therefore likely that phenocopies of these OS linked syndromes are caused by mutations in other genes affecting the same pathways, or that yet undiscovered hypomorphic allelic variants of established susceptibility genes modify tumor risk [9,10]. The presence of such low-penetrance gene variants would imply that a much larger than previously estimated fraction of tumors may be due to a congenital predisposition [11,12]. Since familial clustering would be very unlikely to occur in such conditions [13], population-based genome-wide association studies (GWAS) or whole-genome sequence analysis in patients might help to map the underlying gene variants, but might be hampered by low statistical power. A recent multi-center GWAS study on osteosacoma identified 2 novel candidate loci for OS susceptibility with odds-ratios of 1.57 and 1.39, respectively [14]. The missing association with established OS risk genes such as TP53, RB1, WRN or RECQL4 in this study, however points to the problem of small patient number and genetic and phenotypic heterogeneity for this tumor type.

Strain differences between genetically defined mouse inbred strains have been used successfully to identify genes and allelic variants which influence predisposition for spontaneous or carcinogen-induced malignancies [15-18]. For osteosarcoma, the only established exogenous carcinogen is ionizing radiation, in particular by incorporation of bone-seeking radio-isotopes in the event of medical or occupational exposure. This observation was confirmed in animal models such as dogs and rodents, and variations in the OS susceptibility between different mouse inbred strains were reported [19]. We recently used a mouse model to map several osteosarcoma susceptibility loci in the murine genome using QTL analysis [20,21]. These studies established that the principal susceptibility locus spans an approximately 46 Mbp interval on the distal part of mouse chromosome 14.

The large number of mapped genes and non-coding transcripts in the D14Mit90 to D14Mit225 interval, together with the general problem of deducing a causal relationship from forward genetics, precluded any inference to a single susceptibility gene at this locus. For this purpose, more precise and complementary mapping strategies are necessary to reduce the number of candidate susceptibility genes such that functional studies are feasible. In the present study we combined mapping of tumor-specific allele-losses across the susceptibility locus with positional-candidate mapping and identified a functional gene polymorphism in *Rb1*.

## Results and discussion

### Identification of candidate genes

**Figure 1:**
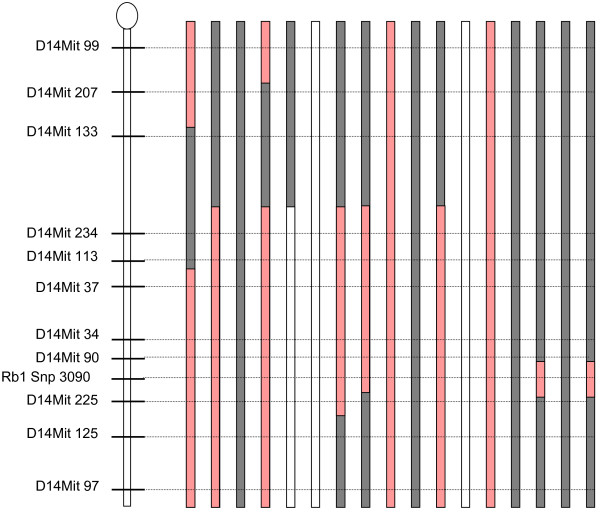
**Analysis of allelic imbalance in 17 osteosarcomas induced in (BALB/cHeNhg x CBA/Ca)F1 hybrid mice.** Pattern of allelic imbalances at the *Rb1* locus and at flanking microsatellite markers are shown as grey bars (retention of heterozygosity), pink bars (reduction or loss of the BALB allele) or white bars (reduction or loss of the CBA allele).

We have previously shown that an interval on murine chromosome Mmu 14 (72.3 – 118 Mbp) contains the principal locus conferring susceptibility to alpha-radiation induced osteosarcoma in four different mouse strains [20,21]. (compound LOD score 4.4). Due to incomplete penetrance of such a susceptibility gene, confounding causes of death after irradiation, and the inherent stochastic nature of radiation tumorigenesis a more precise mapping of this QTL using linkage-analysis was not feasible. We have therefore applied a complementary method to better define the position of the susceptibility gene, using somatic allelic imbalances mapping across this locus in osteosarcoma of (BALB/cHeNhg × CBA/Ca)F1 mice. After 227Thorium incorporation at the age of 100 days, 17 out of 80 female F1 mice developed osteosarcoma that were suitable for molecular analysis (median latency time = 553 days, range from 290 to 766 days). In 13 out of 17 of these tumors we observed allelic loss or imbalance for at least one marker between D14Mit234 and D14Mit97, i.e. within the principle OS susceptibility locus on chromosome 14 [21] (Figure 1). In 10/13 cases the maternal (BALB/c) allele was affected, whereas the paternal CBA allele was lost in the remaining 3 cases. This preferential loss of 10 BALB/c versus 3 CBA alleles (p = 0.014, relative to a total of 34 parental chromosomes) is consistent with the assumption that tumors in heterozygous F1 gain a growth advantage when they somatically lose an allele that confers relative resistance. We observed before that in the progeny of (BALB/cHeNhg × CBA/Ca) F1 mice backcrossed to female BALB/cHeNhg (further referred to as BBC) the BALB/c allele at this locus is associated with relative OS resistance [21]. The smallest common interval of overlapping allelic imbalances spans 2.654 Mbp (between D14Mit90 and D14Mit 225, Figure 1) and encompasses 12 known genes, 1 protein-coding gene with unknown function, 2 non-coding RNA genes and 1 pseudogene (Additional file 1). To nominate candidate genes from this genomic interval, we used PosMed, a search engine based on the entire biomedical literature to find links between a set of genes and a given phenotype [22]. Here we identified 7 genes in the D14Mit90/D14Mit225 interval (*Rb1, Esd, Cysltr2, Htr2a, Fndc3a, Ltm2b, Lpar6*) with a potential function in osteosarcoma susceptibility (Additional file 2). Of these 7 genes, only *Rb1* is directly associated with osteosarcoma as suggested by 12 studies (p = 5 × 10^−53^) whereas the other 6 candidates are linked to osteosarcoma indirectly, either via additional genes acting *in-trans* or through comorbidity (next best p-value 1.1 × 10^−20^ for *Esd*). It is also worth mentioning that according to the Mouse Genome Informatics database, some modifier loci for other traits have been mapped to this interval (such as *Modor3* for ocular retention, *Myci2* for myocardial infarction, *Hwq1* for heart weight). But since they are not defined as transcribed sequences, we limited our screen to genes and other transcribed elements as validated in the current mouse genome release (NCBI GRCm38).

The link between RB1 and osteosarcoma is mainly based on missense and nonsense mutations in the germline which predispose for retinoblastoma in children and OS in adolescents [23]. Following high-dose radiotherapy for primary retinoblastoma in these patients, the risk of a secondary OS is potentiated [24]. We therefore analysed the pattern of allelic imbalances or loss-of-heterozygosity (LOH) across this minimal deleted interval, using polymorphic microsatellite markers and an intragenic SNP at the *Rb1* 3’UTR (Additional file 3). LOH breakpoints were found between the distal (D14Mit225) markers and *Rb1* in three tumors, two of which also had breakpoints in the proximal interval between D14Mit90 and *Rb1* (Figure 1).

### Expression of candidate genes

We further reasoned that any susceptibility gene for osteosarcoma must be expressed in the relevant target cells in order to influence the tumor risk. We therefore measured the mRNA expression in murine osteoblasts of all genes and none-coding elements as derived from the current release of the mouse genome in this interval (Additional file 1). We found that except for three of them with undetectable expression (*Nudt15, Med4, miRNA687hom*), the relative expression level in the MC3T3 preosteoblastic cell-line and in-vitro explanted osteoblasts varied between 0.15 fold (*Cysltr2*) and 3.7 fold (*Ltm2b*) relative to whole embryo mRNA (Additional file 4).

### Rb1 promoter variations

The classical view of genetic predisposition by a germline loss-of-function RB1 allele followed by somatic losses of the wt allele during progression of retinoblastoma is that eventually the tumor expresses the inherited allele which confers high susceptibility in the germline.

We therefore expected to find a gene variation in the CBA allele of *Rb1* which can explain the association with the higher OS risk. Sequencing the coding region of *Rb1* from the BALB/cHeNhg and CBA/Ca strain, we found only nucleotide variations in the 3’ UTR, but as they did not map to known mRNA stability motifs they were not considered of major functional relevance [25]. Missense and non-sense RB1 mutations, which account for about 80% of heritable retinoblastoma and osteosarcoma cases in patients [26-28], can therefore be ruled out in our case. In addition to coding sequence RB1 mutations associated with highly penetrant phenotypes in humans, a small subset of cases exhibit retinoblastoma predisposition with incomplete penetrance, sometimes caused by mutations that affect RB1 promoter methylation sites [9,29-31]. An association of these low-penetrance gene mutations with OS predisposition, however, was never found, but might have been missed due to the scarcity of such families and the general low OS incidence.

**Figure 2:**
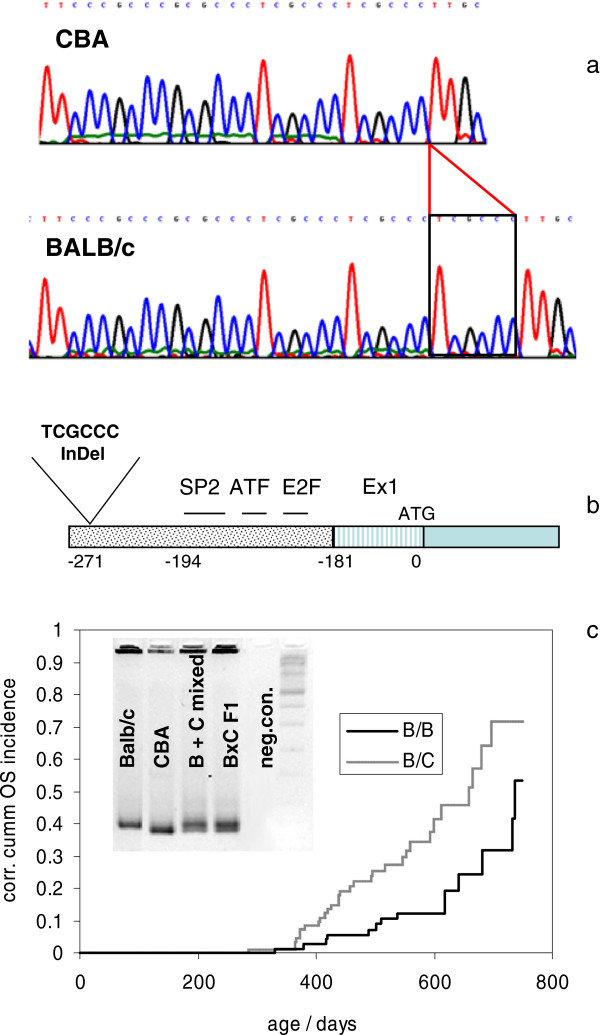
***Rb1 *Promoter polymorphism between BALB/cHeNhg and CBA/Ca mice. a**: Sequence of the *Rb1* promoter region between - 267 nt and – 288 nt determined for strains BALB/cHeNhg and CBA/Ca , showing structure of the BALB specific TCGCCC insertion. Base numbering relative to ATG in exon 1. **b**: Position of the BALB specific hexanucleotide insertion relative to the SP1, ATF and E2F core binding sites according to [32]. **c**: Association of the *Rb1* promoter genotype in BALB/cHeNhg x (BALB/cHeNhg x CBA/Ca) backcrossed mice with osteosarcoma induction. Kaplan-Meier plot of osteosarcoma appearance in 169 BALB/cHeNhg x (BALB/cHeNhg x CBA/Ca) backcrossed mice grouped according to their genotypes at the *Rb1* promoter. Mice inheriting the B/C genotype have a significantly shorter latency time and higher overall osteosarcoma incidence than mice with the B/B genotype (p = 0.0007, Log-Rank Test). Of 79 mice inheriting the B/B genotype 14 developed an osteosarcoma, whereas out of 90 mice with the B/C genotype 30 developed an osteosarcoma. Insert: PCR products following amplification of a 122/128 bp fragment (primer 1005f and 1128r) encompassing the site of the BALB/c specific insertion. The insertion specific fragment was used for genotyping all mice of the backcrossed cohort

In fact, we found a CBA/Ca specific TCGCCC hexanucleotide deletion in the *Rb1* promoter, located 271 nt upstream of the ATG start and 78 nt upstream of the binding sites for Sp1, ATF and E2F in the promoter core (Figure 2a,b) [32]. *In-silico* analysis using the tool MathInspector [33,34] to analyze changes in TF binding sites predicted that this hexanucleotide InDel could delete a WT1 TF binding site and disrupt a SP1-SP1 module (Additional file 5) [35].

### Impact on Rb1 promoter activity and gene expression

**Figure 3:**
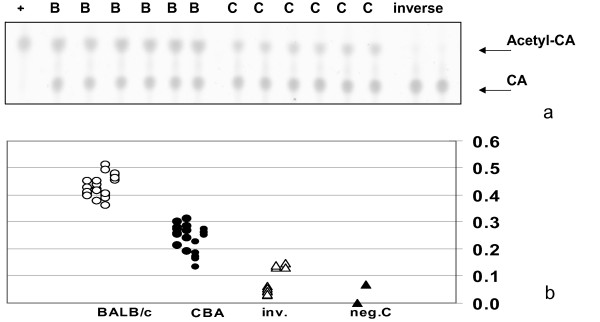
**Analysis of transcriptional activity of the variant allele of the *Rb1 *promoter. a**, CAT activity as measured by TLC chromatography of ROS17/2.8 rat osteoblast protein extracts following transient transfection with the BALB/c and the CBA variants of Rb1 promoter fused to CAT reporter constructs. **b**, Quantification of the CAT reporter signal driven by BALB/c (B) and CBA (C) promoter variants (positive orientation), CBA promoter (inverse orientation) and mock-transfected cells (negC). Transfection efficiency was normalized by simultaneous measurement of luciferase expression from co-transfected RFBpGL3 constructs

To test the impact of these predicted TF binding site alterations on gene expression, we applied both an in-vitro promoter reporter assay as well as direct quantification of *Rb1* transcripts in whole embryos of BALB/cHeNhg and CBA/Ca mouse strains. *Rb1* promoter fragments encompassing the ubiquitous SP1, ATF and E2F binding sites together with the site of the Δ(TCGCCC) variation from both mouse strains (Additional file 6) were able to activate a CAT reporter gene in an orientation-dependent and reproducible manner (Figure 3a) after transient transfection of ROS17/2.8 rat osteoblasts. Quantification of the reporter activity from the two promoter variants confirmed that the CBA–specific Δ(TCGCCC) deletion produced less CAT-activity (2.42 AU, CI 2.19 – 2.65) when compared to the BALB/c allele (4.38 AU, CI 4.19 – 4.57, Figure 3b). This 1.8 fold difference is highly significant (p = 0.0003, t-test). An inverse orientation of the *Rb1* promoter fragment shows only 21% activity and extracts from mock-transfected cells have less than 8.3% basal activity relative to the BALB/c construct. A difference was also found in the average *Rb1* gene expression of day 16 whole embryo extracts, being lower in CBA/Ca (183 AU, CI 138 – 228) than in the BALB/cHeNhg strain (232 AU, CI 165 – 299, p = 0.038, t-test, Additional file 7). Whereas the 1.28 fold reduced mRNA expression in whole-embryo might be a more realistic value in view of the natural genomic context of the *Rb1*-gene, the 1.8 fold lower promoter activity in transfected osteoblasts could reflect some bone-specific regulation of the *Rb1* promoter.

It is interesting to note that although the essential TF-binding sites in the core promoter of the *Rb1* gene are not affected by this alteration, suggesting that transcription is still possible, two *Rb1* control elements map within a 20 bp interval around this hexanucleotide indel [36]. Recently it was shown that these RCEs and other undiscovered sequence elements in-cis of the RB1 core promoter site are crucial for transcriptional fine-tuning in a tissue-specific manner. They are also involved in regulating the *RB1* protein homeostasis through feedback loops involving all 3 Rb family proteins members, including a RB1 autosuppression [37].

If for practical purposes we consider a 1.5 fold lower *Rb1* expression in homozygous CBA/Ca osteoblasts (i.e. the average between the reporter assay and the in-vivo embryo mRNA level), then the overall *Rb1*-expression from both alleles in heterozygous BALB/CBA osteoblasts would be reduced to about 83% as compared to 100% in homozygous BALB/BALB osteoblasts (after loss of the BALB/c allele in OS, expression in malignant osteoblasts could further drop to approximately 67%).

### Δ(TCGCCC) Rb1 promoter variant and the risk for Th227 induced OS

The impact of this reduced *Rb1* expression in normal osteoblast cells for the risk to develop osteosarcoma after Th227-incorporation can be inferred from re-genotyping the BALB/cHeNhg × (BALB/cHeNhg × CBA/Ca) backcross [21] for the *Rb1* promoter variant (Figure 2c). Out of 169 backcrossed mice, 93 inherited the BALB/CBA genotype at the *Rb1* promoter, of which 23 (or 24.7%) developed an OS. Of the 76 mice inheriting the BALB/BALB genotype, only 10 were diagnosed with a tumor (13.2%, p = 0.016, Fisher’s exact test). In addition to increased tumor incidence, the Δ(TCGCCC) promoter variant in the CBA-allele is also linked to a shorter tumor latency (p = 0.0007, Log-Rank test) (Figure 2c).

### The potential impact of reduced RB1 expression for the carcinogenic process

The canonical function of RB1 as a prototype tumor-suppressor gene is based on its ability to regulate the G1/S cell-cycle transition by sequestration of E2F-transcription factors. In bilateral retinoblastoma, this gatekeeper function is completely abolished after loss-of-heterozygosity in carriers of a RB1 germline mutation. In osteosarcoma, although frequently affected by RB1 allelic losses [38], complete absence of gene expression is rarely seen, indicating that a residual function of the wt RB1 protein is compatible with or maybe even accelerates OS growth (Additional file 8). A residual expression was also shown in the majority of other cancer cell lines and tumors except retinoblastoma [26,39], suggesting that reduced expression, but not complete absence of RB1 promotes tumorigenesis

Apart from the canonical RB1 function as a key cell cycle regulator, recent studies also point to its involvement in non-canonical pathways such as regulation of pancentromeric heterochromatin [40], mitotic checkpoint control [41] and telomere maintenance [42,43]. We have recently shown that *Rb1* haplo-insufficiency results in a more rapid telomere attrition and a higher number of anaphase-bridges and micro-nuclei after gamma irradiation in murine osteoblasts [43].

One could make the point that these non-canonical functions of RB1 are all involved in the maintenance of genome integrity during cell division. An impaired function of either of these mechanisms could lead to the accumulation of genetic or chromosomal defects, in particular those which are transmitted from parental to daughter cells. A defect in the canonical RB1 cell-cycle regulation, on the other hand might disturb the growth rate of a cell itself, but not necessarily increase genomic instability in the progenitor cells. A transmission of chromosomal instability from parent to daughter cells due to an impaired RB1 function was also suggested by discovering its involvement in the regulation of centromeric heterochromatin, a key factor for a proper chromosome segregation during mitosis [44]. In particular the genotoxic stress by ionizing irradiation would require an efficient cellular surveillance mechanism for genetic and chromosomal stability to minimize the risk of malignant transformation [45].

Most epidemiological studies indicate that ionizing irradiation can increase the number of solid tumors and leukaemia, without changing the age distribution of their diagnosis. This suggests that the rate of accumulating additional genetic defects during tumor latency is a cell inherent process and only marginally influenced by radiation. The latency time, however shortens dramatically in the event of a congenital tumor predisposition, therefore causing a rise in tumors in children or young adulthood. Regarding their pathobiology, radiation-induced OS with latency times between 10 and 20 years therefore represent tumors of the older patient group. But in combination with an increased genetic susceptibility caused by an inherited low expressing *RB1* allele, these tumors might shift to younger age groups.

We hypothesize that a reduced RB1 expression renders normal cells more vulnerable to secondary genetic changes, and that the resulting loss of genome stability is a driving factor for tumorigenesis in itself. *Rb1* haploinsufficiency in murine ES cells was shown to increase the rate of genetic instability [46], demonstrating that a reduced gene expression can easily compromise genome maintenance. Such a reduced capability of cells to adequately respond to chromosomal defects could have severe consequences after radiation exposure, considering that radiation-induced genotoxicity is mainly due to the generation of DNA strand breaks.

### Structural variants in the human RB1 promoter

**Figure 4:**
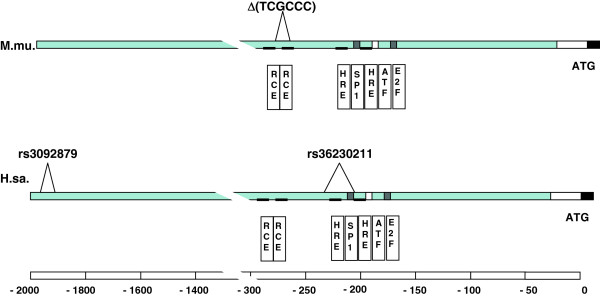
**Binding sites for transcription factors and regulatory proteins in the murine and the human Rb1 promoter (below the line) and position of the reported indel polymorphisms (above line).** TF binding sites in murine sequence as predicted by MatInspector software [33,34] and as annotated by Zacksenhaus [32] (Genebank Acc.-No. M86180). TF binding sites in human sequence as predicted by [36]. Promoter core consisting of steroid hormone response elements (HRE) and Sp1, ATF and E2F binding sites are highly conserved. Retinoblastoma control elements (RCE) in the human promoter are according to [36] and predicted in the murine promoter using published RCE core sequences [48].

The majority of RB1 germline mutations found in congenital retinoblastoma patients cause partial or complete loss of the gene (41%) or frame shift and nonsense mutations (42%) [26-28]. The remaining cases, some of which show incomplete penetrance of the condition, are assumed to carry germline mutations affecting regulatory elements of the RB1 gene. It would therefore be worth systematically testing non-syndromic OS patients for the presence of low-penetrance RB1 promoter variants. Recent efforts to screen the human genome for common variants have shown that the RB1 promoter not only harbours several SNPs, but at least two InDel variants. A hexanucleotide variation (rs2092879) 1.5 kbp upstream of the human RB1 promoter core was shown to alter TF binding and promoter activity in vitro [47], whereas a 23bp InDel variant (rs36230211) is located at a similar distance to the transcription start (221 bp proximal) as the murine InDel variation described here (Figure 4). Considering that the human 23bp InDel removes part of the SP1 site from the core promoter, and that the structure of transcription factor binding sites in this region is highly conserved from mouse to human [32,36] one can assume that carriers of the human InDel variant are also affected by a reduced RB1 expression. The observations that a sufficient RB1 expression is instrumental for cells to prevent structural chromosomal defects from being transmitted to the next cell generation suggests, that a reduced RB1 level might result in a steady accumulation of mutations and genomic instability in normal cells. This would cause a higher incidence of OS in the group of older patients, but at the same time become critical in the event of a genotoxic stress as caused by radiation. It might therefore be interesting to search for the presence of the above mentioned human *RB1* promoter variants in older OS patients and in those presenting with a post-radiation, therapy associated tumor.

## Conclusion

This is the first experimental demonstration of a functional and genetic link between reduced *Rb1* expression from a common promoter variant and increased risk for osteosarcoma after radiation exposure in mice. We propose that a reduced *Rb1* expression by common variants in regulatory regions can modify the risk for a malignant transformation of bone cells after radiation exposure.

The low phenotypic penetrance of such promoter or regulatory variants spares them from significant selection pressure, and hence increases their chance of becoming fixed and more common in the population. We have shown in this study that in combination with exogenous genomic stress like ionizing radiation these low penetrance variations in *RB1* can significantly alter the tumor susceptibility of an organism.

## Methods

### Mouse breeding and tumor induction

All animal experiments were approved by the state government (license no. RegOB 211-2531-49/00 and 211-2531-56/06) and carried out in accordance with the federal animal welfare guidelines. (BALB/cHeNhg × CBA/Ca)F1 and F1 mice backcrossed to female BALB/cHeNhg (further referred to as BBC) mice were bred from HMGU Neuherberg (formerly GSF) breeding stocks. For tumor induction, 80 (BALB/cHeNhg × CBA/Ca)F1 and 169 BBC mice (all female) were injected i.p. at the age of 100 days with a single dose of 185Bq/g ^227^Th (as Thorium Citrate) [19]. Mice were housed 5 to a cage and examined 5 days a week for the development of tumors or other life-threatening conditions. Bone tumors were diagnosed radiologically at necropsy followed by histological examination after EDTA decalcification. All malignant diseases were classified by trained clinical and veterinary pathologists, using H&E and van-Gieson staining. Typical histologies and X-ray images of tumors are deposited in the PATHBASE database (http://eulep.pdn.cam.ac.uk, Acc.No. 3410-3414, Acc.No. PB94713 ff).

### Statistical evaluation of osteosarcoma induction

Osteosarcoma-free survival curves, corrected for all competing causes of death, were calculated using the Kaplan-Mayer method. Significance of differences were tested with the Statistika 6.0 software (StatSoft, Tulsa OK) using Cox’s F- and LogRank-Test for tumor-free survival, two-tailed Fisher’s exact test for fraction of tumor-bearing mice, one-tailed Chi-square test of a four-field contingency table for the allelic imbalances and t-test for the gene-expression and reporter assays.

### DNA extraction, microsatellite genotyping and *Rb1* sequencing

DNA for genotyping of mice was extracted from tail-tips and from macro-dissected tumor-sections for measuring allelic imbalances. Genomic PCR was done using primers for polymorphic microsatellite markers and for the Δ(TCGCCC) *Rb*1 promoter variant (Additional file 9). Reactions were carried out in 96 well plates using a GeneAmp 9700 thermocycler (Applied Biosystems, Foster City, Ca, USA) followed by 90min electrophoresis on 1.5% or 3% agarose/TBE gels.

### Analysis of allelic imbalance and LOH in osteosarcoma

Allelotyping of 17 osteosarcomas diagnosed in (BALB/cHeNhg × CBA/Ca)F1 mice and of the paired normal tissue was done for polymorphic microsatellite markers on chromosome 14 (see above) by allel-specific PCR and for a SNP in the *Rb1* 3’ UTR (3090nt) by genomic sequencing. PCR-amplified microsatellite markers were run for 90min electrophoresis in 3% agarose/TBE/EtBr gels, the PCR products were visualized under UV illumination, captured as 8 bit TIF-files on an electronic DocuGel V system (Mitsubishi, Japan) and quantified using the software package Intelligent Quantifier (BioImage, Jackson MI). To validate linearity and sensitivity of this assay, standard curves of the allelic ratios were generated from BALB/CBA genomic DNA containing different percentages of pure BALB/c or pure CBA/Ca DNA (Additional file 10).

### Candidate positional mapping using PosMed

The smallest common region affected by allelic imbalances in osteosarcoma from (BALB/cHeNhg × CBA/Ca)F1 mice as defined by flanking microsatellite markers was submitted to a semantic web association study using PosMed [22]. The search parameters were set as: search=“gene”, condition=“genomic interval”, species=“mouse”, keyword=“osteosarcoma susceptibility” genome sequence version=“NCBIm37”. search settings=“all documents”. With a cut-off p-value of 0.01 (with Bonferroni correction), the list of gene hits was ranked in the order of increasing p-value.

### qRT-PCR measurement of mRNA expression

mRNA (1μg per sample) was extracted from the MC3T3-E1 preosteoblastic cell line (ATCC code CRL-2593™) and from primary murine osteoblasts grown *in-vitro*[43] using RNAeasy mini kit (Quiagen GmbH, Hilden, Germany) and reverse transcribed using SuperscriptIII (Life Technologies, Carlsbad, CA). Quantitative RT-PCR was done in 96well plates on a StepOne machine using Fast Sybr Green (both Life Technologies, Carlsbad, CA) and gene specific primers (Additional file 9). Quantification of expression levels was done using the ΔΔCt method with a house keeping gene (TBP) and whole embryo cDNA as a calibrator.

#### *Rb1* mRNA northern blot

Whole embryo RNA was extracted from day 16 p.c. BALB/cHeNhg and CBA/Ca embryos by homogenisation in liquid N_2_ followed by GTC extraction and phenol/chloroform purification. For each strain, 6 embryos from 3 different pregnant mice were used. Following northern blot of 10μg of each RNA sample, membranes (Roche Diagnostics, Germany) were probed with a 630 bp ^32^P labelled mouse *Rb1* cDNA fragment spanning exon 10 to 13. Signals were quantified using a Storm scanner (Amersham Pharmacia, Uppsala, Sweden) and Image Quant software (Molecular Dynamics, Sunnyvale CA, USA). Relative expression levels (AU) are given as the integrated optical densities of the *Rb1* signal normalized to the sum of the 28S and 18S RNA signals.

#### Prediction of promoter function

A 352bp sequence containing the putative *Rb1* promoter from BALB/cHeNhg and CBA/Ca mice, including known upstream regulatory sequences, the 5’-UTR and the initiation codon ATG was analysed for potential TF binding sites using MatInspector software (Genomatix Software GmbH, Munich, Germany) [33,34]. Predefined transcription factor weight matrix descriptions are based on the TRANSFAC-database (all-vertebrates library) with the following default parameter: core similarity = 0.75 and matrix similarity = calculated optimised.

#### CAT reporter assay

A fragment of the *Rb*1 promoter was PCR amplified from CBA/Ca (451 bp) and BALB/cHeNhg (457 bp) genomic DNA, using primers 6F and 8.2B, Pwo-polymerase (Peqlab, Erlangen, Germany) and reaction conditions as given in Additional file 9. The fragment was cloned into TOPO TA® vector (Life Technologies, Carlsbad, CA) and directionally sub-cloned into the pCAT3 basic vector (Promega Corp. Madison WI, USA) using SacI and XhoI restriction sites. Plasmids BALB 36, CBA 11 and CBA 14–5 (Additional file 6) were purified using Quiagen plasmid MaxiPrep kit (Quiagen GmbH, Hilden, Germany) yielding a final concentration of 2 μg/μl and OD260/OD280 absorption ratios between 1.68 and 1.73. A luciferase expression vector pGL3® (Promega Corp., Madison WI, USA) under the control of the RFB LTR served as an internal control for transfection efficiency. ROS 17/2.8 osteoblasts at a density of 5*10^5^ cells per 60mm plate were transiently transfected with 2μg Rb1pCAT and 0.2μg pGL3 using FuGene® lipofection (Roche Diagnostics GmbH, Mannheim, Germany). After 48h, cells were harvested using reporter lysis buffer (Promega Corp, Madison WI, USA) and 20μg protein extracts were assayed for CAT activity using the FastCAT yellow® assay ((Life Technologies Molecular Probes, Carlsbad, CA). After thin-layer chromatography, TLC plates (Sigma Aldrich, Germany) were visualized on a Storm laser scanner (Amersham Pharmacia, Uppsala, Sweden) and quantified using Image Quant software (Molecular Dynamics, Sunnyvale CA, USA). Luciferase expression was measured using the luciferase assay system (Promega Corp. Madison WI, USA) and an Auto-Lumat LB953 luminometer (EGG Berthold, Pforzheim, Germany). Promoter activity was expressed in arbitrary units as AU = 1000* ac-CA/((CA + ac-CA)* AU_luc_), wherein CA and ac-CA are the integral densities of the chloramphenicol and the acetyl-CA signal, respectively, and AU_luc_ is the luciferase signal.

### Data access

Histological X-ray images of representational cases of osteosarcoma were deposited to PATHBASE database (http://eulep.anat.cam.ac.uk, acc.No. PB94713 ff). *Rb*1 promoter sequence variation was deposited to the European Nucleotide Archive (http://www.ebi.ac.uk/ena/) and to the Database of genomic variants archive (http://www.ebi.ac.uk/dgva/, acc.No. pending).

## Abbreviations

AU: Arbitrary units; CA: Chloramphenicol; CAT: Chloramphenicol-acetyl-transferase; DMEM: Dulbecco’s modified Eagle medium; FCS: Fetal calf serum; GTC: Guanidiniumthiocyanate; GWAS: genome-wide association study; i.p.: Intraperitoneal; LOD: logarithm of the odds ratio for linkage; LOH: Loss-of-heterozygosity; OD: Optical density; OR: Odds-ratio; OS: Osteosarcoma; QTL: Quantitative trait locus; RCE: Retinoblastoma control element; RFB-LTR: Long terminal repeat of the RFB virus; SNP: Single nucleotide polymorphism; STS: Sequence tagged site; TLC: Thin layer chromatography; TSG: Tumor suppressor gene; TF: Transcription factor; UTR: Untranslated region.

## Competing interests

The authors declare that none of the data in this study are affected by competing interests of a third party.

## Authors’ contributions

(MR) planned and conducted the animal studies, cloning and reporter assay of the promoter variant, (IGV) measured expression of candidate genes after mapping by PosMed in normal osteoblasts and RB1 in human OS cell lines, (TD) did *Rb1 mRNA* measurements on microdissectied murine tumors, (VK) performed the LOH/allelic imbalance studies on F1 tumors, (RS) did the in-silico promoter modelling using MathInspector, (MK) performed the tumor histo-pathology, (JF) helped with haplotype analysis and genetic mapping of the murine locus, (MN) provided data on SNP LOH and RB1 mutations of human OS, (MJA) supervised the study and contributed to discussion and conclusion of the paper. All authors read and approved the final manuscript.

## Supplementary Material

Additional file 1 List of annotated genes on mouse chromosome 14, 72 600 000 Mbp - 75 300 000 Mbp, their function, expression in osteoblasts and evidence for an association with osteosarcoma by PosMed-search. Click here for file

Additional file 2 **Search for a candidate gene for osteosarcoma susceptibility using the online mapping and annotation tool PosMed.** The interval on murine chromosome 14 between D14Mit90 (72.6 Mbp) and D14Mit225 (75.3 Mbp) was analyzed for genes for which literature records have a co-citation with the key-word “Osteosarcoma Susceptibility”). Co-citation of Rb1 with “Osteosarcoma Susceptibility” in 12 publications occurs with highest significance (p = 5 × 10–53), whereas four other genes are only indirectly co-cited with the phenotype and have lower significance (p < 1.2 × 10–20). Click here for file

Additional file 3 **Strain polymorphism in the 3’ UTR used for genotyping tumor vs. normal tissue.** In the lower panel two representative cases with loss of the BALB- and CBA-alleles are shown, resp. Click here for file

Additional file 4 **mRNA expression of mapped candidate genes at the OS susceptibility interval in normal osteoblasts and in a MC3T3 pre-osteoblast cell line.** Expression level is relative to TBP housekeeping mRNA. All values are mean +/−2xSE from 3 independent osteoblast cultures. Click here for file

Additional file 5 **Position of transcription factor binding sites within the Rb1 promoter sequence as predicted using MatInspector software.** The binding sites above the sequence (in black letters) are present in the BALB / CBA consensus sequence, whereas binding sites below the sequence (in red letters) are unique for the BALB allele. Asterisks show TF binding sites as annotated by Zacksenhaus et al. 1993 (Genebank Acc.-No. M86180). Click here for file

Additional file 6 **Reporter constructs for in-vitro analysis of Rb1 promoter activity using CAT reporter constructs fused to 452/458 bp long fragments derived from the BALB/c and CBA-variant of the Rb1 promoter.** As negative control, an inverse orientated CBA-fragment was used. Base numbering is according to Genebank Acc.-No. M86180. Click here for file

Additional file 7 ***Rb1*-mRNA expression in BALB/cHeNhg vs. CBA/Ca mouse embryos 16 days p.c. as measured by northern blot.** Click here for file

Additional file 8 **Expression of RB1-protein in established human OS cell lines (S8a), of Rb1 mRNA in 21 human primary osteosarcoma in comparison with OS cell lines U2OS (Rb1 positive) and SaOS (Rb1 deficient) (S8b) and *Rb1 *mRNA in murine OS cell lines (S8c).** Note that with a few exceptions, all murine and human osteosarcoma maintain a low expression of RB1 mRNA and protein expression. Click here for file

Additional file 9 Primer Sequences and PCR conditions for genomic and qRT-PCR. Click here for file

Additional file 10 **Protocol for the quantification of allelic imbalance/loss-of-heterozygosity using genomic PCR of strain - specific microsatellite markers.** Quantitative loss or imbalances of BALB- or CBA alleles was calibrated for each of the used markers. For this purpose, pure BALB/c and pure CBA/Ca genomic DNA were mixed in different ratios and used as template for PCR (27, 30, 33 and 35 cycles). After running the PCR products on agarose gel electrophoresis and recording the pattern of EtBr stained BALB- and CBA bands, densitometric measurement of the two signals was done. The ratio of the experimentally determined BALB- and CBA alleles was compared with the relative amount of BALB- and CBA-DNA mixed in the PCR templates and served to draw calibration curves for the later measurement of allelic ratios in the tumor DNA. Click here for file
