# Supplementary material for: A Rb1 promoter variant with reduced activity contributes to osteosarcoma susceptibility in irradiated mice
Source: Mol Cancer. 2014 Aug 4;13:182. doi: 10.1186/1476-4598-13-182 (PMC4237942; doi:10.1186/1476-4598-13-182)
Supplement: Additional file 1 — List of annotated genes on mouse chromosome 14, 72 600 000 Mbp - 75 300 000 Mbp, their function, expression in osteoblasts and evidence for an association with osteosarcoma by PosMed-search. [file 1476-4598-13-182-S1.pdf]

| | Gene Name | Pos / Mbp | OB Expression | PosMed Search | Funktion |
|---|---|---|---|---|---|
| D14Mit90 | | 72.276 | | | |
| | Fndc3a | 72.5 - 72.7 r | xx | x | fibronectin type III domain containing 3A |
| | Cysltr2 | 73.05 r | x | x | cysteinyl leukotriene receptor 2 |
| | Rcbtb2 | 73.2 f | xxx | | regulator of chromosome condensation (RCC1) and BTB (POZ) domain containing protein 2 |
| | Rb1 | 73.18 r | xx | xxx | Retinoblastoma susceptibility |
| | miRNA687(homol) | 73.2 | o | | micro RNA |
| | Lpar6 | 73.24 f | xx | | lysophosphatidic acid receptor 6 |
| | Ltm2b | 73.36 r | xxx | | integral membrane protein 2B |
| | Med4 | 73.5 f | o | | mediator of RNA polymerase II transcription, subunit 4 homolog (yeast) |
| | Nudt15 | 73.5 r | o | | nudix (nucleoside diphosphate linked moiety X)-type motif 15 |
| | Sucla2 | 73.5 f | xx | | succinate-Coenzyme A ligase, ADP-forming, beta subunit |
| | AC155246.1 | 73.6 f | x | | unknown, protein coding |
| | AC131029.1 | 73.7 | x | | snRNA |
| | Gm6984 | 73.9 r | o | | pseudogene |
| | Htr2a | 74.6 - 74.7 f | xxx | x | 5-hydroxytryptamine (serotonin) receptor 2A |
| | Esd | 73.7 f | xxx | x | esterase D/formylglutathione hydrolase |
| | Lrch1 | 74.7 - 74.9 r | xxx | | leucine-rich repeats and calponin homology (CH) domain containing 1 |
| D14Mit225 | | 74.930 | o: < 0.01<br>x: 0.01 - 0.1<br>xx: 0.1 - 1<br>xxx: > 1 | | |

**Table S2:** List of annotated genes on mouse chromosome 14, 72 600 000 Mbp - 75 300 000 Mbp, their function, expression in osteoblasts and evidence for an association with osteosarcoma by PosMed-search.
