# Supplementary material for: A Rb1 promoter variant with reduced activity contributes to osteosarcoma susceptibility in irradiated mice
Source: Mol Cancer. 2014 Aug 4;13:182. doi: 10.1186/1476-4598-13-182 (PMC4237942; doi:10.1186/1476-4598-13-182)
Supplement: Additional file 2 — Search for a candidate gene for osteosarcoma susceptibility using the online mapping and annotation tool PosMed. The interval on murine chromosome 14 between D14Mit90 (72.6 Mbp) and D14Mit225 (75.3 Mbp) was analyzed for genes for which literature records have a co-citation with the key-word “Osteosarcoma Susceptibility”). Co-citation of Rb1 with “Osteosarcoma Susceptibility” in 12 publications occurs with highest significance (p = 5 × 10^–53^), whereas four other genes are only indirectly co-cited with the phenotype and have lower significance (p < 1.2 × 10^–20^). [file 1476-4598-13-182-S2.pdf]

Home User's guide

PosMed Positional Medline

search: gene | condition: genomic interval | species: mouse
Select Interval with OmicBrowse | chromosome: 14 | position from 72600000 to 75300000 | clear | version: NCBIm37
keyword: osteosarcoma susceptibil | search | clear | gene name: | show: 20 | ▸search settings

Mouse gene hits

download rank list
set as target
check all

1. Rb1
2. Esd
3. Itm2b
4. Cysltr2
5. Lpar6
6. Htr2a
7. Fndc3a

Total Hits: 7 (1.084 sec)

display mode: Simple

1. Rb1 , retinoblastoma 1 Score: 1.04E-50077

	Name	No. of docs	P value	Position
	**Rb1**	4088 docs		Mm:14:73595309-73725547
		18 docs	1.04E-77	
	**osteosarcoma susceptibility**			

2. Esd , esterase D/formylglutathione h... Score: 1.81E-50020

	Name	No. of docs	P value	Position
	**Esd**	438 docs		Mm:14:75132179-75149655
		2 docs	1.81E-20	
	**osteosarcoma susceptibility**			

3. Itm2b , integral membrane protein 2B Score: 1.81E-30020

	Name	No. of docs	P value	Position
	**Itm2b**	84 docs		Mm:14:73762219-73785096
		30 docs	5.35E-46	
	**Alzheimer disease**	86841 docs		
		2 docs	1.81E-20	
	**osteosarcoma susceptibility**			

4. Cysltr2 , cysteinyl leukotriene receptor... Score: 1.81E-30020

	Name	No. of docs	P value	Position
	**Cysltr2**	164 docs		Mm:14:73428935-73448879
		62 docs	2.12E-75	
	**Asthma**	132457 docs		
		1 doc	1.81E-20	
	**osteosarcoma susceptibility**			

5. Lpar6 , lysophosphatidic acid receptor... Score: 1.81E-30020

	Name	No. of docs	P value	Position
	**Lpar6**	46 docs		Mm:14:73637807-73639587
		11 docs	4.28E-25	
	**Mrgpra3**	10237 docs		Mm:7:54844320-54856742
		1 doc	1.81E-20	
	**osteosarcoma susceptibility**			

6. Htr2a , 5-hydroxytryptamine (serotonin... Score: 1.81E-30020

	Name	No. of docs	P value	Position
	**Htr2a**	4171 docs		Mm:14:75040647-75106666
		656 docs	1.43E-492	
	**Dopamine**	119859 docs		
		1 doc	1.81E-20	
	**osteosarcoma susceptibility**			

7. Fndc3a , fibronectin type III domain co... Score: 1.79E-30013

	Name	No. of docs	P value	Position
	**Fndc3a**	29 docs		Mm:14:72937753-73109810
		6 docs	1.79E-13	
	**Fn1**	30448 docs		Mm:1:71632097-71699745
		4 docs	9.31E-20	
	**osteosarcoma susceptibility**			

Please send comments and questions to omicspace@gmail.com
Copyright © RIKEN (The Institute of Physical and Chemical Research), Japan. All rights reserved. Our site policy.

1. Gonzalez-Vasconcellos I, Anastasov N, Sanli-Bonazzi B, Klymenko O, Atkinson MJ, Rosemann M: **Rb1 haploinsufficiency promotes telomere attrition and radiation-induced genomic instability**. *Cancer Res* 2013, **73**(14):4247-4255.
2. Collard TJ, Urban BC, Patsos HA, Hague A, Townsend PA, Paraskeva C, Williams AC: **The retinoblastoma protein (Rb) as an anti-apoptotic factor: expression of Rb is required for the anti-apoptotic function of BAG-1 protein in colorectal tumour cells**. *Cell Death Dis* 2012, **3**:e408.
3. Gonzalez-Vasconcellos I, Domke T, Kuosaite V, Esposito I, Sanli-Bonazzi B, Nathrath M, Atkinson MJ, Rosemann M: **Differential effects of genes of the Rb1 signalling pathway on osteosarcoma incidence and latency in alpha-particle irradiated mice**. *Radiat Environ Biophys* 2011, **50**(1):135-141.
4. Flowers S, Beck GR, Jr., Moran E: **Transcriptional activation by pRB and its coordination with SWI/SNF recruitment**. *Cancer Res* 2010, **70**(21):8282-8287.
5. Sabado Alvarez C: **Molecular biology of retinoblastoma**. *Clin Transl Oncol* 2008, **10**(7):389-394.
6. Wang A, Schneider-Broussard R, Kumar AP, MacLeod MC, Johnson DG: **Regulation of BRCA1 expression by the Rb-E2F pathway**. *J Biol Chem* 2000, **275**(6):4532-4536.
7. Smith E, Frenkel B, MacLachlan TK, Giordano A, Stein JL, Lian JB, Stein GS: **Post-proliferative cyclin E-associated kinase activity in differentiated osteoblasts: inhibition by proliferating osteoblasts and osteosarcoma cells**. *J Cell Biochem* 1997, **66**(2):141-152.
8. De Chiara A, T'Ang A, Triche TJ: **Expression of the retinoblastoma susceptibility gene in childhood rhabdomyosarcomas**. *J Natl Cancer Inst* 1993, **85**(2):152-157.
9. Hinds PW, Mittnacht S, Dulic V, Arnold A, Reed SI, Weinberg RA: **Regulation of retinoblastoma protein functions by ectopic expression of human cyclins**. *Cell* 1992, **70**(6):993-1006.
10. Wunder JS, Czitrom AA, Kandel R, Andrulis IL: **Analysis of alterations in the retinoblastoma gene and tumor grade in bone and soft-tissue sarcomas**. *J Natl Cancer Inst* 1991, **83**(3):194-200.
11. Templeton DJ, Park SH, Lanier L, Weinberg RA: **Nonfunctional mutants of the retinoblastoma protein are characterized by defects in phosphorylation, viral oncoprotein association, and nuclear tethering**. *Proc Natl Acad Sci U S A* 1991, **88**(8):3033-3037.
12. Hansen MF: **Molecular genetic considerations in osteosarcoma**. *Clin Orthop Relat Res* 1991(270):237-246.
13. Zhu XP, Dunn JM, Phillips RA, Goddard AD, Paton KE, Becker A, Gallie BL: **Preferential germline mutation of the paternal allele in retinoblastoma**. *Nature* 1989, **340**(6231):312-313.
14. Cavenee WK: **Chromosomal mechanisms in the initiation of human familial mixed cancers**. *Princess Takamatsu Symp* 1986, **17**:171-179.
