# Supplementary figures and images for: A Rb1 promoter variant with reduced activity contributes to osteosarcoma susceptibility in irradiated mice
Source: Mol Cancer. 2014 Aug 4;13:182. doi: 10.1186/1476-4598-13-182 (PMC4237942; doi:10.1186/1476-4598-13-182)

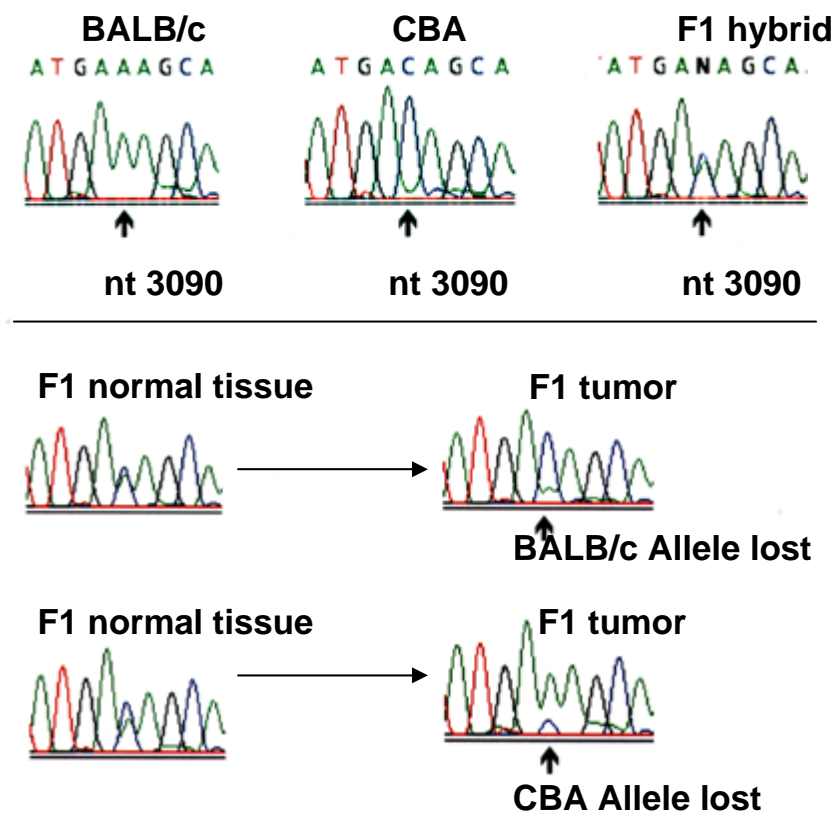

Fig S1

Supplement: Additional file 3 — Strain polymorphism in the 3’ UTR used for genotyping tumor vs. normal tissue. In the lower panel two representative cases with loss of the BALB- and CBA-alleles are shown, resp. [file 1476-4598-13-182-S3.pdf]

### Rel Expression in Osteoblasts

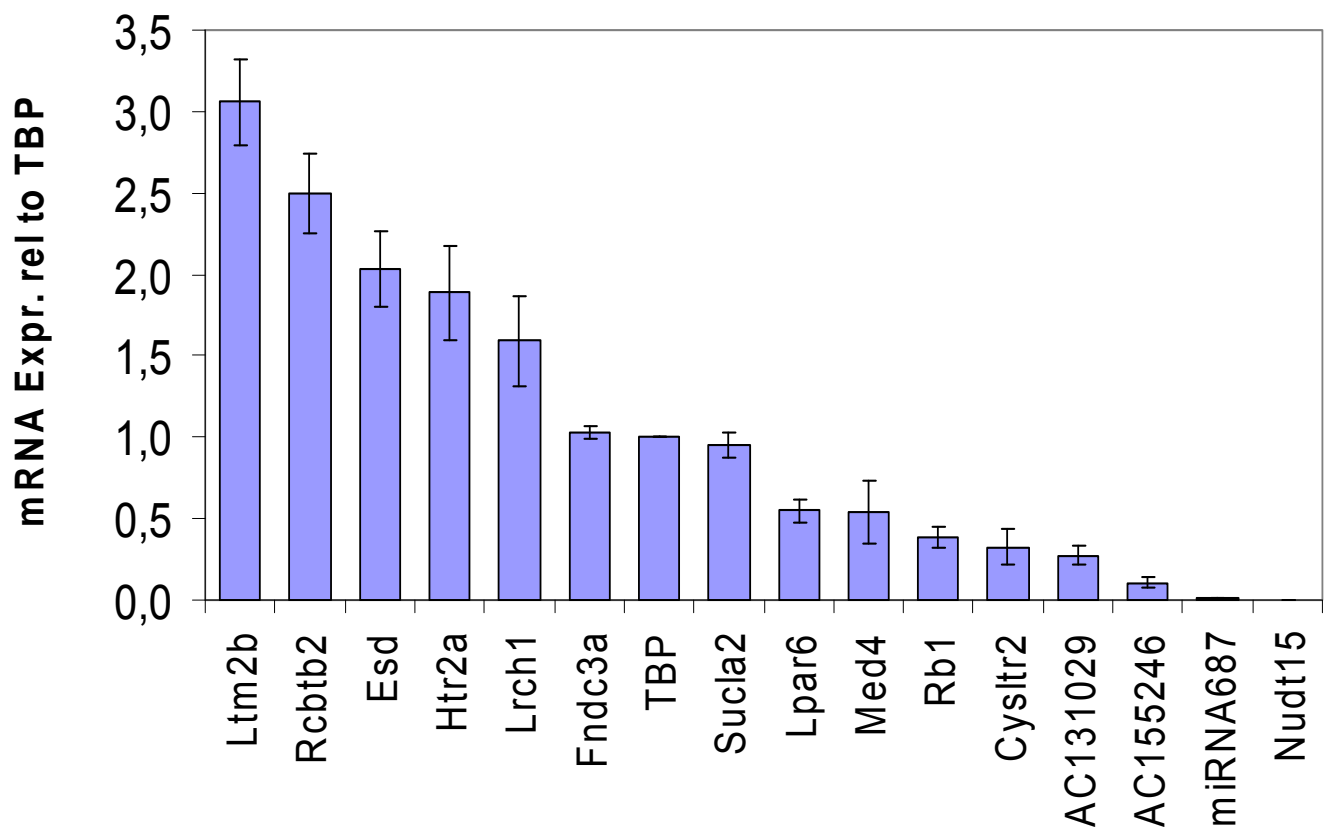

Supplement: Additional file 4 — mRNA expression of mapped candidate genes at the OS susceptibility interval in normal osteoblasts and in a MC3T3 pre-osteoblast cell line. Expression level is relative to TBP housekeeping mRNA. All values are mean +/−2xSE from 3 independent osteoblast cultures. [file 1476-4598-13-182-S4.pdf]

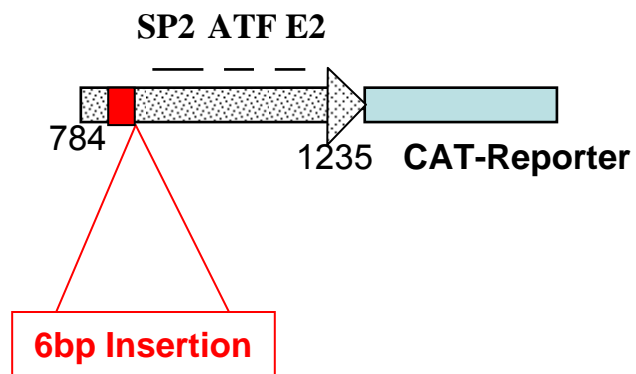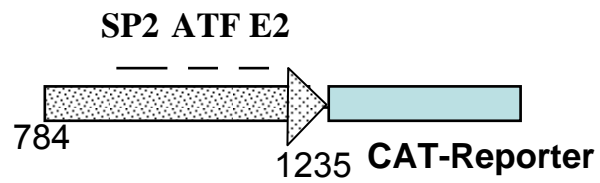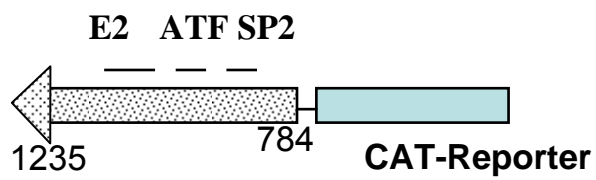

Suppl 7

Supplement: Additional file 6 — Reporter constructs for in-vitro analysis of Rb1 promoter activity using CAT reporter constructs fused to 452/458 bp long fragments derived from the BALB/c and CBA-variant of the Rb1 promoter. As negative control, an inverse orientated CBA-fragment was used. Base numbering is according to Genebank Acc.-No. M86180. [file 1476-4598-13-182-S6.pdf]

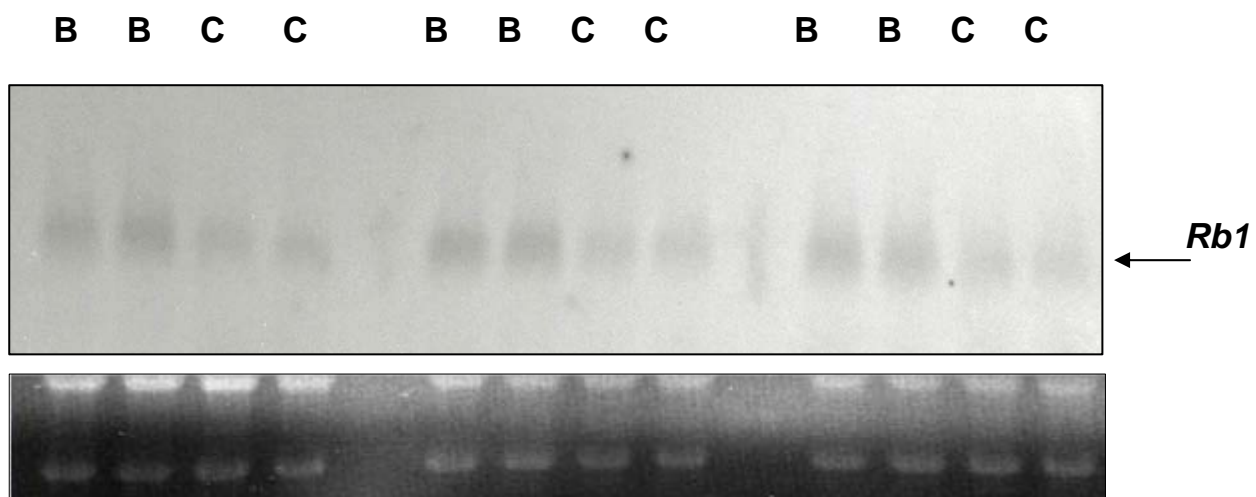

Suppl 5

Supplement: Additional file 7 — Rb1 -mRNA expression in BALB/cHeNhg vs. CBA/Ca mouse embryos 16 days p.c. as measured by northern blot. [file 1476-4598-13-182-S7.pdf]
