# Supplementary material for: A Rb1 promoter variant with reduced activity contributes to osteosarcoma susceptibility in irradiated mice
Source: Mol Cancer. 2014 Aug 4;13:182. doi: 10.1186/1476-4598-13-182 (PMC4237942; doi:10.1186/1476-4598-13-182)
Supplement: Additional file 5 — Position of transcription factor binding sites within the Rb1 promoter sequence as predicted using MatInspector software. The binding sites above the sequence (in black letters) are present in the BALB / CBA consensus sequence, whereas binding sites below the sequence (in red letters) are unique for the BALB allele. Asterisks show TF binding sites as annotated by Zacksenhaus et al. 1993 (Genebank Acc.-No. M86180). [file 1476-4598-13-182-S5.pdf]

```
                                                          WT1
                                                E2F
                                                                  WT1
                                                   SP1
-330
CBA     acaggcccgg  gcaggcgcgg  cttccccgcg  gcttcccgcc  cgcgccctcg  ccctcgccc------c
BALB    acaggcccgg  gcaggcgcgg  cttccccgcg  gcttcccgcc  cgcgccctcg  ccctcgccctcgcccc
                                            SP1 – SP1 Modul
                                                                     WT1

                                 SP1
                                 SP1                   ATF
-270
CBA     ttgcccgccg  ccgccgccac  cgccgccccg  gttccccaac  tgacgcccgc  ggcgcgagcc
BALB    ttgcccgccg  ccgccgccac  cgccgccccg  gttccccaac  tgacgcccgc  ggcgcgagcc

                                   AP1
                                        AP1
          AP1F                                     E2F
                                         ATF
-210                       SP1*           ATF*           E2*
CBA     tcgcggacgt  gagcgcgggc  ggaagtgacg  ttttcccgcg  gttggcccgg  ggctcggttg
BALB    tcgcggacgt  gagcgcgggc  ggaagtgacg  ttttcccgcg  gttggcccgg  ggctcggttg
```

Suppl 6
