# Supplementary material for: A Rb1 promoter variant with reduced activity contributes to osteosarcoma susceptibility in irradiated mice
Source: Mol Cancer. 2014 Aug 4;13:182. doi: 10.1186/1476-4598-13-182 (PMC4237942; doi:10.1186/1476-4598-13-182)
Supplement: Additional file 8 — Expression of RB1-protein in established human OS cell lines (S8a), of Rb1 mRNA in 21 human primary osteosarcoma in comparison with OS cell lines U2OS (Rb1 positive) and SaOS (Rb1 deficient) (S8b) and Rb1 mRNA in murine OS cell lines (S8c). Note that with a few exceptions, all murine and human osteosarcoma maintain a low expression of RB1 mRNA and protein expression. [file 1476-4598-13-182-S8.pdf]

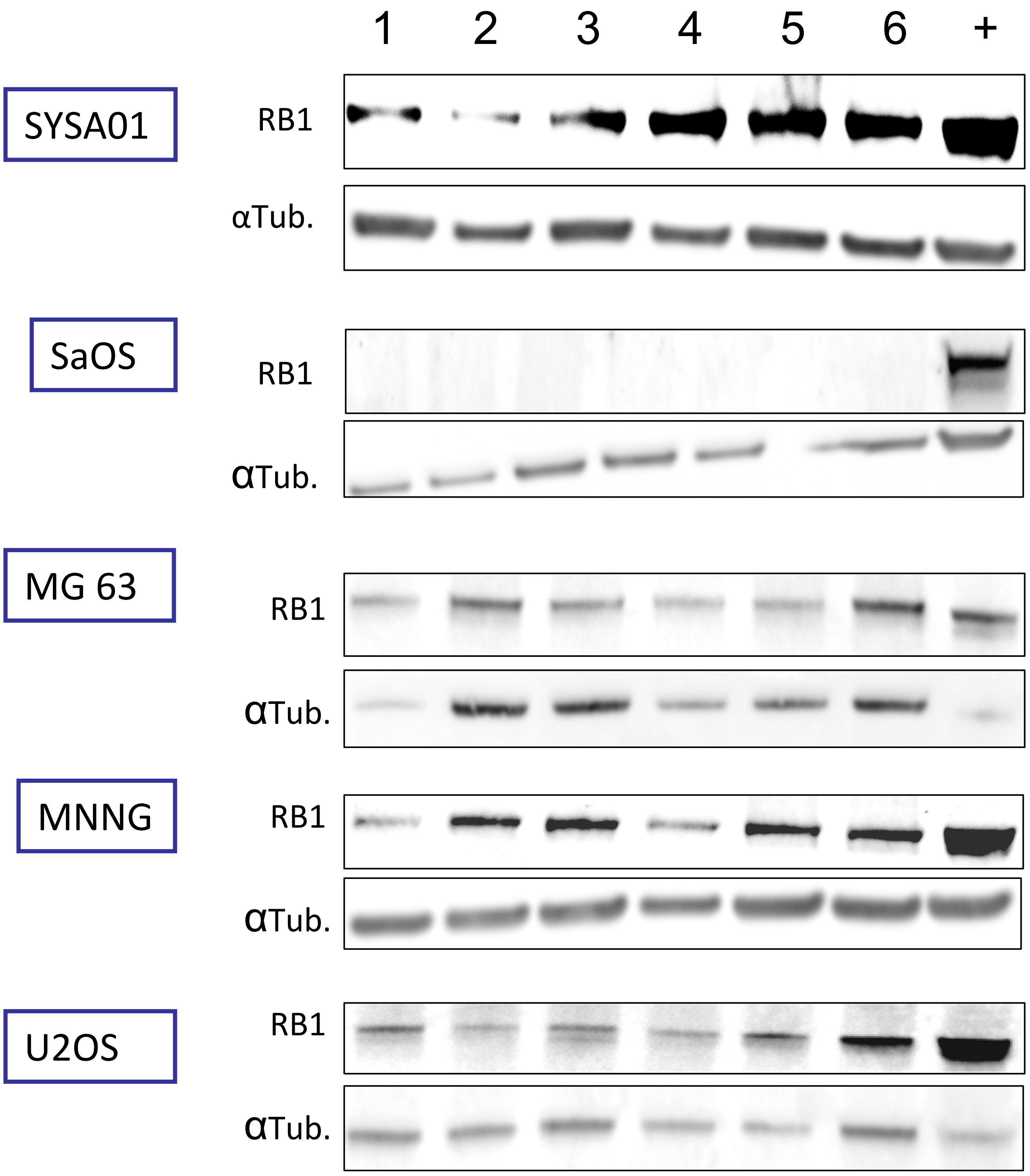

S8a: RB1 protein expression in human osteosarcoma cell lines

20µg protein extracts from 5 unirradiated (1-3) or 4Gy gamma-irradiated (4-6) human OS cell lines were probed on western-blot for RB1 (mAB G3-245, BD Pharmingen) and α-Tubulin as loading control. Protein extracts were harvested 0h (1,4), 2h (2,5) or 4h (3,6) after treatment. + Positive control: protein extracts from HEK293 cells.

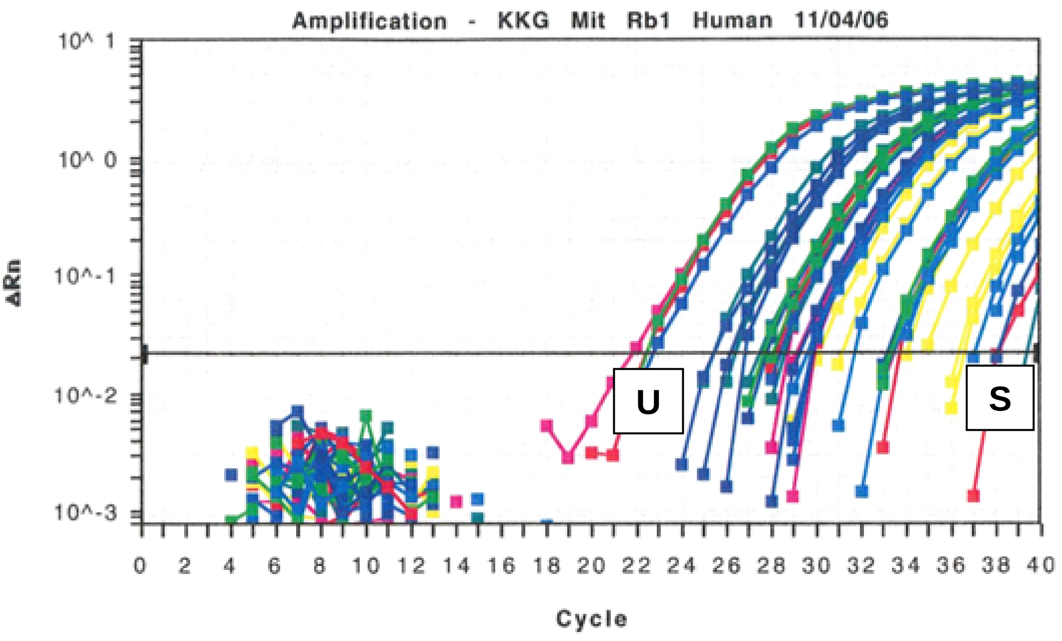

Case	Rel. mRNA
OS-1	2,17
OS-2	0,25
OS-3	3,66
OS-4	1,58
OS-5	0,64
OS-6	0,17
OS-7	1,02
OS-8	1,04
OS-9	0,34
OS-10	0,09
OS-11	0,69
OS-12	4,36
OS-13	0,21
OS-14	0,27
OS-15	0,20
OS-16	1,44
OS-17	0,32
OS-18	0,52
OS-19	0,68
OS-20	0,17
OS-21	0,19
SaOS	0,00
U2OS	1,11
Cal	1,00

S8b: RB1 mRNA Expression in human osteosarcoma

RB1 cDNA amplification plot and rel. mRNA expression of 21 primary human Osteosarcoma cases in comparison with cell line U2OS (**U**, expressing Rb1) and SaOS (**S**, Rb1 deficient).

After reverse transcription using SuperSript III (Invitrogen) cDNA from tumors and cell-lines were amplified using the real-time quantitative system (Taq.Man) ABI PRISM 7700 SDS (Applied Biosystems) for RB1 (probe Hs00153108_m1 and TBP (probe Hs00427620_m1). Expression values are relative to TBP and normalized to a Calibrator (pooled human cell lines)

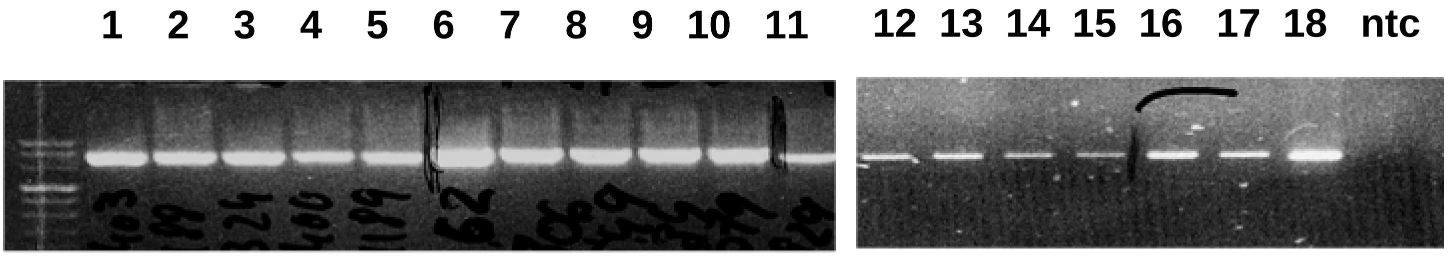

mRb1 Rt-PCR using primers Ex2f-363 and Ex11r-1231 to amplify a 887 bp long fragment from cDNA derived from murine osteosarcoma cell lines 1403, 1188, 1324, 1400, 1189, 62, 306, 549, 734, 679, 1929, 182, 733, 734 (1-11,16-18) and total embryo mRNA from CBA/CA (12-15) + no template control.

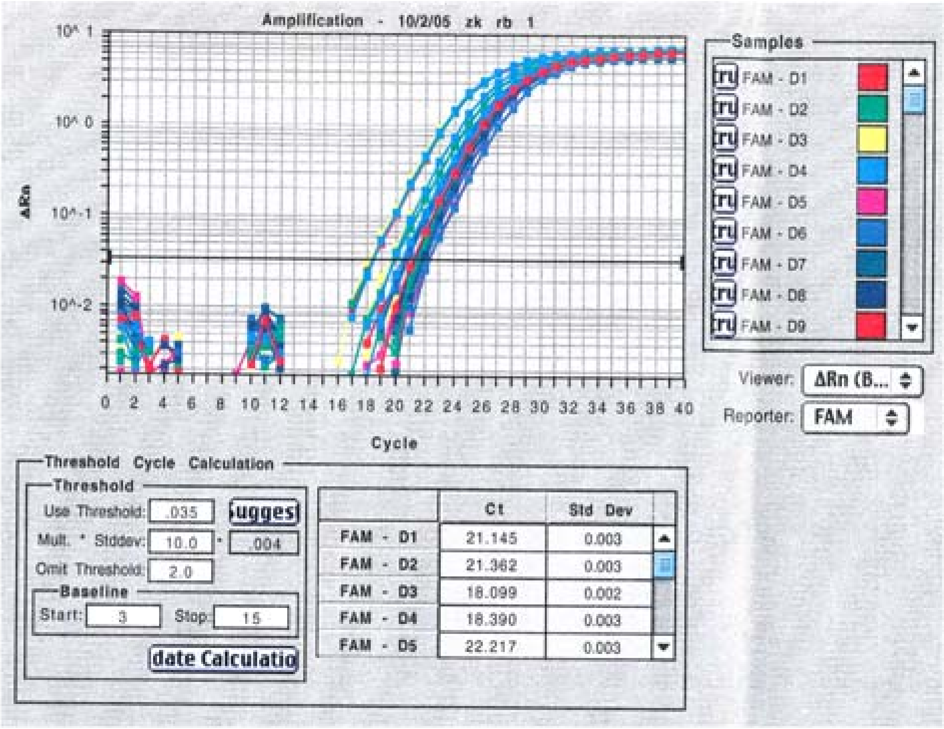

Cell Line	Rb1 rel to TBP to embryo
MOS62	0,32
MOS162	0,68
MOS184#15	0,46
MOS184#20	0,39
MOS306	0,27
MOS549	0,22
MOS679	0,11
MOS733	0,14
MOS734	0,06
MOS1189	0,98
MOS1324	0,18
MOS1400	0,15
MOS1929	0,23
MC3T3	0,09
Whole Embryo	1,00
ntc	<< 0.0001

S8c: Rb1 mRNA expression in murine osteosarcoma cell lines

Rb1 cDNA amplification plot and rel. mRNA expression of 13 murine Osteosarcoma cell lines, mesenchymal cell line MC3T3 in comparison with whole-embryo mRNA.

After reverse transcription using SuperSript III (Invitrogen) cDNA was amplified using the real-time quantitative system (Taq.Man) ABI PRISM 7700 SDS (Applied Biosystems) for mouse *Rb1 (*probe Mm01310561_m1) and *Tbp* (probe Mm00446973_m1). *Rb1* Expression values are relative to TBP and normalized to a Calibrator (whole embryo).
