# Supplementary material for: A Rb1 promoter variant with reduced activity contributes to osteosarcoma susceptibility in irradiated mice
Source: Mol Cancer. 2014 Aug 4;13:182. doi: 10.1186/1476-4598-13-182 (PMC4237942; doi:10.1186/1476-4598-13-182)
Supplement: Additional file 9 — Primer Sequences and PCR conditions for genomic and qRT-PCR. [file 1476-4598-13-182-S9.pdf]

Primer	Sequence	PCR Conditions
mRb-flox-212	GAAAGGAAAGTCAGGGACATTGGG	Ta = 58 ^{o}C
mRb-flox-18	GGCGTGTGCCATCAATG	
mCreCol_f	CCTGGAAAATGCTTCTGTCCGTTTGCC	Ta = 62 ^{o}C
mCreCol_r	GAGTTGATAGCTGGCTGGTGGCAGATG	
mRb1_p6_F	TCTGTGCGTGACTCGCCCATC	Ta = 64 ^{o}C, +4% DMSO
mRb1_p8.2_R	GGAACGTCCCCCGAGGAAAA	
mRb1-Ex17_F	CAAAGTGGAAGCCAACTTGA	Ta = 60 ^{o}C (qRT-PCR)
mRb1-Ex17_R	CACTTACTGAAAGCCATGCAA	
mRb1-Ex19_F	TGAAATCTACCTCCCTTGCC	Ta = 60 ^{o}C (qRT-PCR)
mRb1-Ex19_R	CTGGAGCCACAACTTAACCTAGT	
Fndc3a F	AGATGCCCACTCTACACACG	Ta = 60 ^{o}C (qRT-PCR)
Fndc3a R	TTCCTTGGCGATCCTTTAAT	
Cysltr2 F	CCTGAATTTGGGAAAGGAAG	Ta = 60 ^{o}C (qRT-PCR)
Cysltr2 R	TCACCATGGAGAGAAGCAAG	
Rcbtb2 F	TTCGTCAGGCTTGTGTCTTC	Ta = 60 ^{o}C (qRT-PCR)
Rcbtb2 R	GTGCTCTGAATGTCGCCTAC	
Rb1 F	TCTCACCTCCTGCACTACTCA	Ta = 60 ^{o}C (qRT-PCR)
Rb1 R	CGCTCCTGTTCTGACCTCTT	
Lpar6 F	GGTCCGACTTGGAACAGTTT	Ta = 60 ^{o}C (qRT-PCR)
Lpar6 R	GTCAAGGAAGCCAACCCATA	
Ltm2b F	TTGCTCTTCAGCCAGATGAT	Ta = 60 ^{o}C (qRT-PCR)
Ltm2b R	GAAGGCTCGTTCAGGATGAC	
Med4 F	GCTGCAGAAACAGCTGAAAG	Ta = 60 ^{o}C (qRT-PCR)
Med4 R	TTTCCTTTGCCTGGTAAACA	
Nudt15 F	ATTTGGAATTCGGTGAGACC	Ta = 60 ^{o}C (qRT-PCR)
Nudt15 R	CAGCTTCTTCCCAGGTTTCT	
Sucla2 F	GTCTGTGAGCGCAAATATCC	Ta = 60 ^{o}C (qRT-PCR)
Sucla2 R	GCAGCCACATCTTCAATGTT	
AC155246.1 F	CTAAGGGGGAAACTCATATC	Ta = 60 ^{o}C (qRT-PCR)
AC155246.1 R	CTTGGGTGAATTTGCTTC	
Gm6984 F	TCTCGACCCTGAACGAATAA	Ta = 60 ^{o}C (qRT-PCR)
Gm6984 R	ACTTCATGTTCGCCTCCTTT	
Htr2a F	GAACCAAAGCCTTCCTGAAA	Ta = 60 ^{o}C (qRT-PCR)
Htr2a R	AATCATCCTGTAGCCCGAAG	
Esd F	TCATAAATGCCAATTTCCCA	Ta = 60 ^{o}C (qRT-PCR)
Esd R	ATTGGAGCAAATGCTGACAC	
Lrch1 F	AATTTGCAGATGTTGACCCA	Ta = 60 ^{o}C (qRT-PCR)
Lrch1 R	CTTTGAGAGGCAGACCACAC	

Supplement 9: Primer sequences and PCR conditions for genomic and qRT-PCR
