# Supplementary material for: A Rb1 promoter variant with reduced activity contributes to osteosarcoma susceptibility in irradiated mice
Source: Mol Cancer. 2014 Aug 4;13:182. doi: 10.1186/1476-4598-13-182 (PMC4237942; doi:10.1186/1476-4598-13-182)
Supplement: Additional file 10 — Protocol for the quantification of allelic imbalance/loss-of-heterozygosity using genomic PCR of strain - specific microsatellite markers. Quantitative loss or imbalances of BALB- or CBA alleles was calibrated for each of the used markers. For this purpose, pure BALB/c and pure CBA/Ca genomic DNA were mixed in different ratios and used as template for PCR (27, 30, 33 and 35 cycles). After running the PCR products on agarose gel electrophoresis and recording the pattern of EtBr stained BALB- and CBA bands, densitometric measurement of the two signals was done. The ratio of the experimentally determined BALB- and CBA alleles was compared with the relative amount of BALB- and CBA-DNA mixed in the PCR templates and served to draw calibration curves for the later measurement of allelic ratios in the tumor DNA. [file 1476-4598-13-182-S10.pdf]

4.2.2. Validation of the allelotyping assay for detection of LOH/AI

4.2.2.1. Allele dilution experiments

A pilot study was performed using a panel of DNA samples prepared to mimic LOH/AI. Genomic DNA from BALB/c and CBA mice was mixed together in a series of different ratios to reflect the mixed cell populations expected in tumour biopsies **(Table 4.4.)**. Mixtures were analysed by PCR using four microsatellite markers (D4Mit166, D9Mit154, D11Mit140, and D14Mit125).

Table 4.4. LOH/AI analysis of DNA mixtures mimicking the mixed cell population expected to occur in tumour tissue
Sample 1 contains only BALB/c DNA, presenting tumour DNA with 100% LOH in the uniform tumour cells population. Samples 2 - 10 contain increasing amounts of (BALB/c + CBA) genomic DNA, mixed in a ratio 1:1, reaching mixture expected in the true heterozygote normal tissue (Sample 11). Allelic imbalance in Samples 1 - 10 was estimated as described (see Methods 3.8.1.) in comparison with Sample 11.

Sample number	TUMOUR DNA	NON-TUMOUR DNA	Tumour Contami-nation (%)	Allele (1) in PCR reaction (%)	Allele (2) in PCR reaction (%)	Allelic Imbalance (LOH/AI) (%)
	Relative content of homozygote allele (BALB/c)	Relative content of heterozygote alleles (BALB/c + CBA)				
1	10	0	0	100	0	100
2	9	1	10	95	5	95
3	8	2	20	90	10	89
4	7	3	30	85	15	82
5	6	4	40	80	20	75
6	5	5	50	75	25	67
7	4	6	60	70	30	57
8	3	7	70	65	35	46
9	2	8	80	60	40	33
10	1	9	90	55	45	18
11	0	10	100	50	50	0

Replicate PCR amplifications (n = 3) were performed for a variable number of cycles (27, 30, and 33). The PCR products were visualised by UV-light under different exposure (n = 3) and digitally quantified twice. All markers gave very similar titration curves, and confirmed the observation made by Hu et al. (1996) that different markers are detected with similar sensitivities. The smallest value of the standard deviation was observed at 30 cycles of PCR. **Figure 4.3.** presents the experimental data from the mixed DNA samples for one microsatellite marker (D14Mit125).

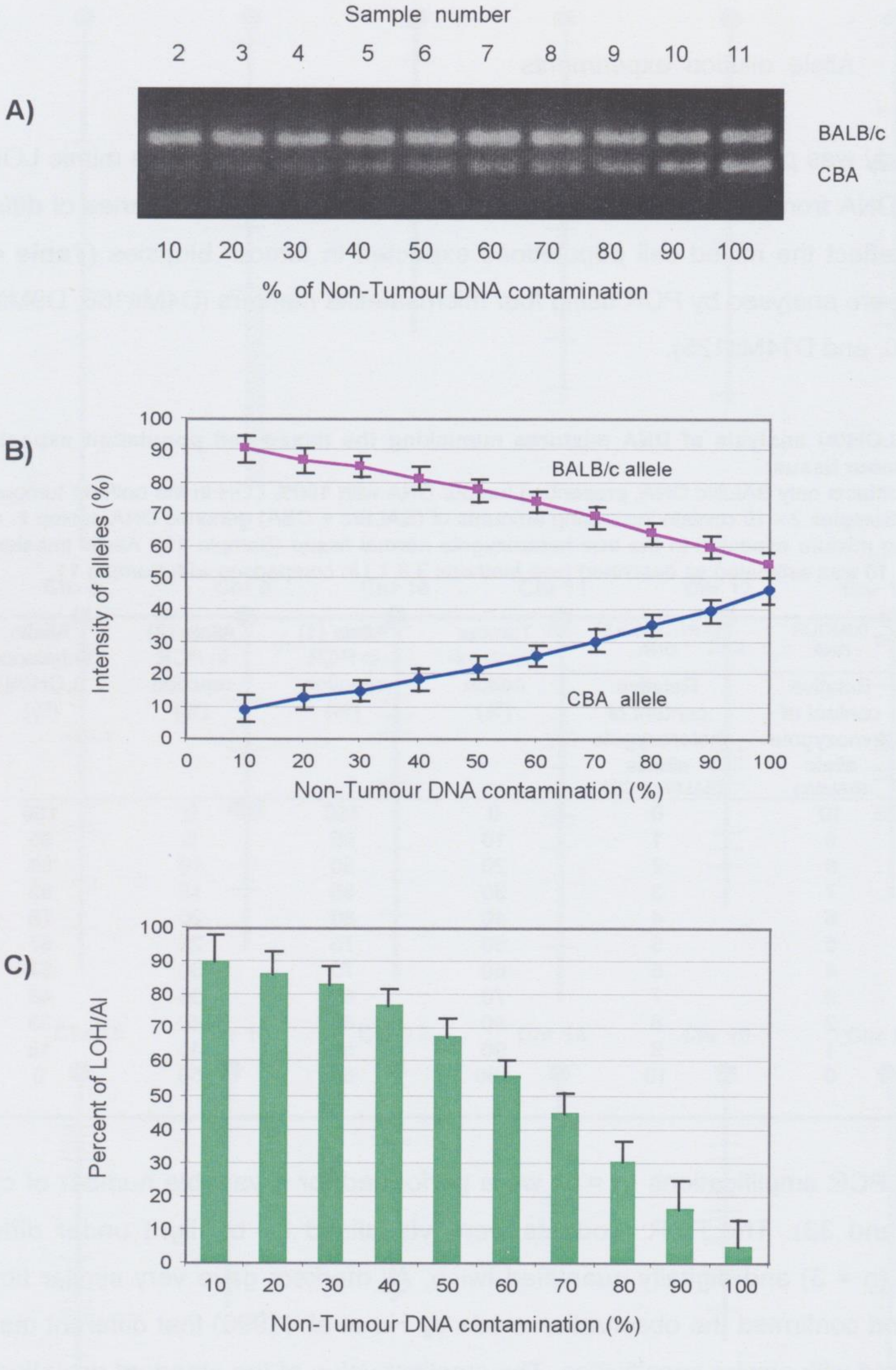

Figure 4.3. PCR analysis of LOH/AI in DNA mixtures
A) 3% agarose gel electrophoresis of PCR products for microsatellite marker D14Mit125.
B) Allele dilution curves at 30 cycles of PCR (n = 3). The figure shows the intensity of the BALB/c and CBA allele at each level of non-tumourous DNA contamination.
C) LOH/AI in the presence of varying proportions of contaminants of non-tumour tissue. LOH/AI (n = 18) in each PCR was estimated in comparison with Sample 11 representing a normal tissue.

4.2.2.2. A 50% cut-off value is selected for definition of LOH/AI

The experimental data (Figure 4.3.) followed the predicted values presented in Table 4.4. **(Figure 4.4.).** In repetitive PCR samples the difference in allelic ratio in Tube 11 mimicking normal tissue was (mean ± 1SD) 4.84 ± 8.1% (see Figure 4.3.C). Reduction of one allele in mixed DNA samples could be detected but not verified in the presence of 90% of non-tumourous DNA (LOH/AI = 16.29 ± 8.62%). The consistent loss of one allele could be detected and validated in DNA samples containing up to 75% of normal cell DNA, corresponding to a 38% LOH/AI. This value was calculated from Figure 4.3.C, where LOH/AI at 70% of normal tissue contaminants was (mean ± 1SD) 44.5 ± 6.18% and at 80% of normal tissue contaminants was 30.4 ± 6.19%. Under these experimental conditions the minimum LOH/AI value of 38.32% (at 70% of normal tissue contaminants) did not overlap with the maximum LOH/AI value of 36.59% (at 80% of normal tissue contaminants), and therefore allowed the assigment of a cut-off value for confident LOH/AI detection. These results are consistent with the published data (LIU et al, 1999).

Nevertheless, for the analysis of actual tumour samples, 50% of allelic imbalance was taken as the conventional cut-off for LOH/AI definition, as used elsewhere (STERN et al., 2000; COOL and JOLICOEUR, 1999; RITLAND et al., 1997; TAO et al., 1996; ZHUANG et al., 1996; WISEMAN et al., 1994). The chosen value of 50% of allelic imbalance at a certain marker means that at least one third of the cell population in the tumour exhibits allelic imbalance at this marker.

In practice a number of markers occasionally showed allelic imbalance between 38% and 50%. The term partial LOH (pLOH) was used to indicate those cases and the LOH/AI was only considered significant when flanking markers showed definitive allelic imbalance, or when the marker mapped to a region within the MAR (Minimum Affected Region, further called as elsewhere MDR, Minimum Deleted Region) flanked by markers showing only background alterations. Even then, these markers were not used to define borders of MDRs.

Allelic imbalance at a marker below 38% was considered to be negative for LOH/AI (see Figure 4.4.), and corresponds to less than one fifth of the cell population affected by LOH/AI.

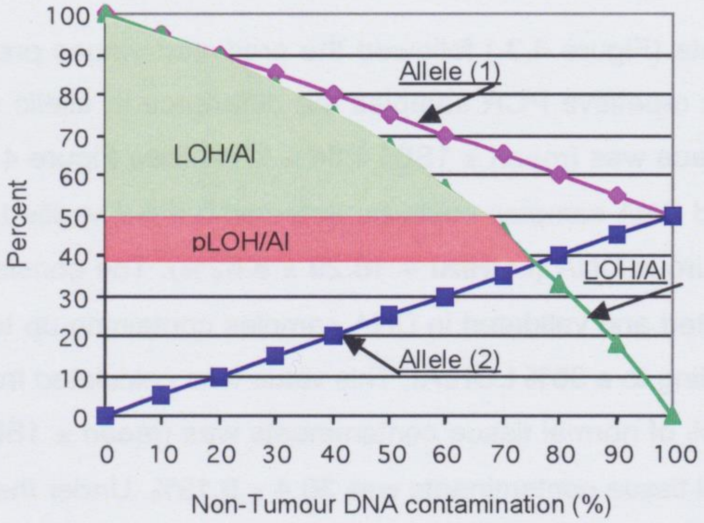

Figure 4.4. Theoretical performance of allelic imbalance detection
The confident zone for LOH/AI detection was divided in two areas: high confidence (green) and low confidence (red). High confidence area represents the interval of contaminants up to 65%, corresponding to LOH/AI values between 100% and 50%. The upper limit of the low confidence interval is 75% of the contaminants. In the low confidence area detection of LOH/AI (pLOH/AI) is ambiguous.
